# Manufacturing a Long-Period Grating with Periodic Thermal Diffusion Technology on High-NA Fiber and Its Application as a High-Temperature Sensor

**DOI:** 10.3390/s18051475

**Published:** 2018-05-08

**Authors:** Xiang Shen, Bin Dai, Yingbin Xing, Luyun Yang, Haiqing Li, Jinyan Li, Jingang Peng

**Affiliations:** Wuhan National Laboratory for Optoelectronics, Huazhong University of Science and Technology, Wuhan 430074, China; shenxiang@hust.edu.cn (X.S.); daibin@hust.edu.cn (B.D.); ybxing@hust.edu.cn (Y.X.); luyunyang@gmail.com (L.Y.); lhq@mail.hust.edu.cn (H.L.); ljy@mail.hust.edu.cn (J.L.)

**Keywords:** fiber optics, fiber optics sensor, long period grating, high-temperature sensor

## Abstract

We demonstrated a kind of long-period fiber grating (LPFG), which is manufactured with a thermal diffusion treatment. The LPFG was inscribed on an ultrahigh-numerical-aperture (UHNA) fiber, highly doped with Ge and P, which was able to easily diffuse at high temperatures within a few seconds. We analyzed how the elements diffused at a high temperature over 1300 °C in the UHNA fiber. Then we developed a periodically heated technology with a CO_2_ laser, which was able to cause the diffusion of the elements to constitute the modulations of an LPFG. With this technology, there is little damage to the outer structure of the fiber, which is different from the traditional LPFG, as it is periodically tapered. Since the LPFG itself was manufactured under high temperature, it can withstand higher temperatures than traditional LPFGs. Furthermore, the LPFG presents a higher sensitivity to high temperature due to the large amount of Ge doping, which is approximately 100 pm/°C. In addition, the LPFG shows insensitivity to the changing of the environment’s refractive index and strain.

## 1. Introduction

Long-period fiber gratings (LPFGs) have been widely used in the fields of optical communication, optical fiber sensing, and lasers [1]. LPFGs are usually manufactured by tapering with a CO_2_ laser [2], inscription with a UV laser [3], or etching with a femtosecond laser [4]. All of these methods introduce a periodic modulation of the refractive index (RI) along the longitudinal axis of the fiber, creating a permanent LPFG. A new type of tunable LPFG can be mechanically induced without damaging the structure [5]. Furthermore, an electrically induced LPFG in a fiber filled with a liquid crystal has been proposed in [6].

The temperature fiber sensor has been widely used in many fields, such as security, health, and industry. Many researchers’ work has focused on increasing the sensitivity of temperature sensors. A Brillouin fiber laser can be used as a temperature sensor that has ultrahigh resolution [7,8]. In this kind of sensor, the high-order Brillouin Stokes process has been used to achieve a high temperature sensitivity. One of the applications of LPFGs is to use them as temperature sensors. As LPFGs manufactured by UV-laser irradiation would be destroyed at high temperatures, they are always used at ambient temperature. An LPFG has been proposed by Davis et al. that was fabricated with a CO_2_ laser [9,10]; the modulation of this kind of LPFG is based on residual stress relief and densification of the glass [10]. It can withstand ultra-high temperature (1200 °C) and has high sensitivity (the sensitivity is about 116 pm/°C from 900 °C to 1200 °C). Conventional LPFGs are usually created on single-mode fibers (SMFs) with a small amount of Ge doping, and the sensitivity is not sufficiently high. Therefore, some researchers have considered changing the material of the fiber, proposing a chemical composite fiber Bragg grating [11] that can withstand much higher temperatures after heat treatment. A type of LPFG in which the elements’ thermal diffusion is based on N-Ge-doped fiber [12] and P-doped fiber [13] has been proposed by Dianov et al., and it can also withstand high temperature (over 1000 °C) [12,13]. A type of LPFG based on B-Ge-doped fiber inscribed with a UV laser has an ultra-high sensitivity to temperature that can reach 2.75 nm/°C [14].

In a similar fashion, we have two goals in fabricating a LPFG for high-temperature sensors. The first is that LPFG be able to withstand high temperature, which requires us to search for a new method for fabricating the LPFG. The second is to find a type of fiber that can cause the LPFG to have high sensitivity to temperature. Thus, we attempt to fabricate a LPFG on a highly Ge-doped fiber, which can easily achieve Ge diffusion and has large thermo-optic coefficients. The modulation of this kind of LPFG is formed by the change of the core’s RI and diameter, which is based on the elements’ diffusion caused by the thermal effect of a CO_2_ laser.

## 2. Materials and Methods

### 2.1. The Point-by-Point Heating Technology for Manufacturing an LPFG in a UHNA Fiber

**Figure 1:**
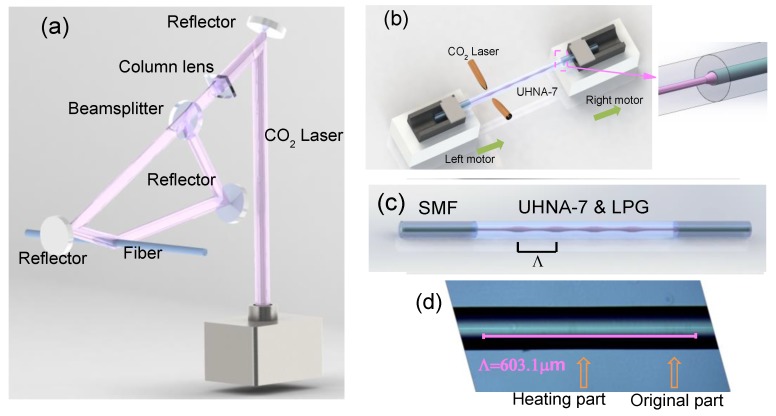
(**a**) The schematic illustration of the optical path in the LZM-100. (**b**) Formation of an LPFG on a UHNA-7 fiber with a CO_2_ laser splicer. (**c**) The structure of the LPFG on the UHNA-7 fiber. (**d**) The side image of the LPFG under a microscope.

The device we used to fabricate the LPFG is a CO_2_ laser splicer (LZM-100, Fujikura, Japan). The optical path of the device is shown in Figure 1a. At first, the laser is divided into two beams, which are at an angle of 170 degrees with a beam splitter. Then, the two beams converge to the same point on the fiber with reflectors, which are shown in Figure 1a. The LZM-100 provides a heating source of which the diameter of the laser spot is about 1 mm. We added a column lens to compress the spot from a circle to an oval, and the width of the spot is 300 μm. With this size of the laser spot, the fiber can be heated without introducing extra damage, such as an etching effect. The inventors of the LZM-100 have proposed a method to manufacture a periodically-tapered LPFG [15]. With the advantages of the device, Masri proposed a new kind of LPFG that has a strong asymmetric structure [16], and it can be used as a mode-selective device. With mechanical oscillations produced by the LZM-100, Shahal has proposed another kind of LPFG that has an off-resonance spectral response [17].

Inspired by these researchers’ work in manufacturing LPFGs by periodically tapering with the LZM-100, we tried to search for another type of LPFG; the core of the fiber can thermally expand under high temperature, it has a changeable mode field diameter (MFD) and NA of the core, and has no changes in the diameter of cladding. This suggests that an LPFG could be formed by periodically heating this fiber. The periodic change in the MFD and NA of the fiber could provide the core modulation for the LPFG, and the period of the modulation is about several hundred micrometers.

**Figure 2:**
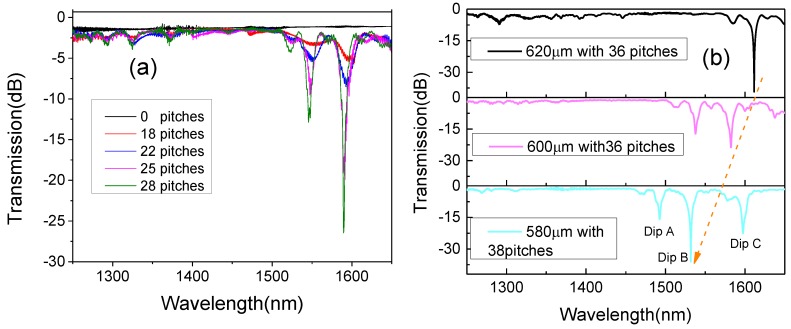
(**a**) Transmission intensities of the LPFG as it forms (for an increasing number of pitches), and (**b**) the intensities of LPFGs with three different pitches.

To create such an LPFG, we chose an ultrahigh-numerical-aperture fiber (UHNA-7, Nufern; the diameters of the core and cladding are 2.4 μm and 125 μm, respectively, and the NA is 0.41). This kind of UHNA fiber is often used as a bridge between SMFs and thin-core fibers (TCF) (or wave-guided devices) [18]. It can be easily fused to an SMF with a standard splicer (62S, Fujikura, Japan) by increasing the discharge intensity to +20 bits and the time to 17,000 ms, which can introduce a transition area (shown in the insert figure of Figure 1b) to reduce the loss caused by the mode field’s mismatch or high-order mode‘s excitation (the standard splicer parameters for fusing SMF to SMF are 0 bit and 2000 ms, respectively). When we fused two pieces of SMF onto each end of the UHNA-7 (the length of the UHNA-7 is about 4.5 cm), it made this structure (SMF-UHNA7-SMF) similar to a conventional sandwich structure (SMF-TCF-SMF). However, due to the special splicing parameter, there is a slight loss and no interference fringes on the spectrum, which is shown in Figure 2a (black line). This is different from the conventional sandwich structure, which usually shows some interference fringes on the spectrum [19], and it can be used as part of a pH sensor [20].

First, we fused an SMF to both ends of a piece of the UHNA-7 fiber. One end of the SMF was connected to an optical spectrum analyzer (OSA; AQ6370D, Yokogawa, Japan) and the other to an optical broadband source (BBS; SLED, 1250–1650 nm). Then, we put the UHNA-7 fiber on a CO_2_ laser splicer.

We set the processing program, which consisted of the following steps, and is shown in Figure 1b:

- Step 1: the two beams of the CO_2_ laser are focused on the same point of the UHNA-7 (Figure 1a) with a power of 400 bits (the actual total power of two beams is 13.3 W) and continues for 9500 ms. At the same time, both right and left motors do not move, which means there is no extra stress being introduced. The LZM-100 provides a continuous heating source, and the spot of the laser is about 300 μm. With this step, one point of the UHNA-7 fiber is heated with a high temperature for 9500 ms.
- Step 2: the left and right motors move in the same direction and speed, which causes the UHNA-7 fiber as a whole to move to the next position. The moving distance is the length of one pitch (e.g., 600 μm). This step is used to determine the length of one pitch of the LPFG.
- Circle: the program jumps from Step 2 to Step 1, then restarts from Step 1, causing Step 1 and Step 2 to create a circle, and we set the number of cycles;
- These steps can be written into the device as a program which is shown in Table 1.

By Step 1, Step 2, and the number of cycles, an LPFG (with a pitch (Λ) of 600 μm) can be automatically produced after we start up the machine. Manufactured with this method, the LPFG has little effect on the external diameter of the UHNA-7 fiber, and the structure of the LPFG on the UHNA-7 fiber is shown in Figure 1c. From the side image of the LPFG, we can find that the core’s image of the original part is a bright line with clear border. In contrast, the core’s image of the heated part does not have a clear border (the zoomed picture will be shown in the Discussion section). The length between the midpoints of the two bright lines is about 603.1 μm, which is similar to a pitch of 600 μm. The formation of the spectrum can be observed in real-time during the manufacture with the OSA. The spectrum of an LPFG with a pitch of 600 μm is shown in Figure 2a. Furthermore, we created several LPFGs with different pitches of 620 μm (black line, 36 pitches), 600 μm (red line, 36 pitches), and 580 μm (green line, 38 pitches), and their spectra are shown in Figure 2b.

In Figure 2a, we can see that the center wavelength of the LPFG shifts to shorter wavelengths as the number of pitches increase during manufacture. The NA decreased as the Ge in the core diffused to the first cladding. As the number of pitches increases, the average RI of the core decreases, similar to what happens in type IIA fiber Bragg gratings [21], which means this kind of grating can be seen as a negative-RI grating. As a result, the effective RI decreases more for the fundamental mode than for the cladding mode. According to Equation (1), Δn decreases, which makes λ (the center wavelength of the LPFG) shift to shorter wavelengths:

(1) $$\lambda =\Lambda \cdot \Delta n=\Lambda \left(n_{eff,fundamental}-n_{eff,cladding}\right)$$

As this type of LPFG is manufactured at high temperature, it was conceivable that it could withstand high temperatures and, thus, be used as a high-temperature sensor. To check if this was true, we put the LPFG in a tube furnace, fixing each end with a three-dimensional adjustment frame to make the LPFG straight. In an initial experiment, we raised the temperature in 100 °C steps. We found that the variation of the center wavelength with temperature was not linear and that the sensitivity was too high, especially for temperatures above 600 °C. After cooling the LPFG to room temperature, the spectra of the heated and unheated LPFGs (at the same temperature) showed large differences. Hence, we presume that some irreversible change happened when heating at temperatures above 600 °C. In this situation, this kind of LPFG cannot be used as a high-temperature sensor unless being subjected to pre-processing.

### 2.2. The Post-Processing for the LPFG to Be Used as a High-Temperature Sensor

**Figure 3:**
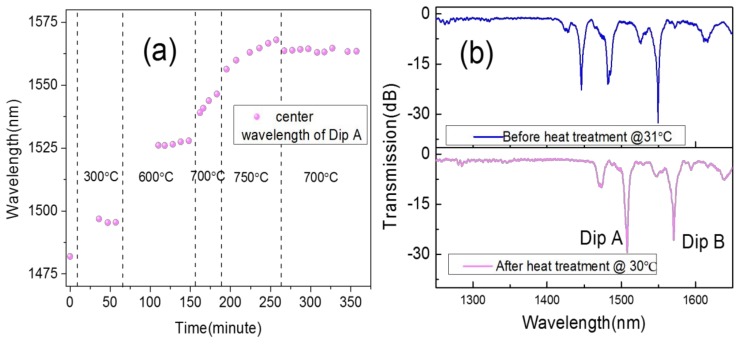
Post-processing of the LPFG. (**a**) The center wavelength shifts with time and increasing temperature. (**b**) Comparison of the spectrum of the LPFG before and after heat treatment.

From Figure 3a, the center wavelength of the LPFG remains shifted to long wavelengths with time at a constant temperature (above 600 °C). We supposed that this was caused by the release of residual stress. The core of UHNA-7 was highly doped with Ge, which means that the melting point is much lower than the cladding. When the UHNA-7 is heated using the point-by-point method, the residual stress of the heated point can be released, but the residual stress of the unheated point still exists. Thus, when the UHNA-7 is under high temperature (above 600 °C), the release of residual stress at the unheated point will increase the average effective RI of the core [9]. From Equation (1), if the effective RI of the core increases, it causes the center wavelength to shift to longer wavelengths at a constant high temperature. Since the higher the temperature, the more residual stress is released, we wondered if we could make the residual stress release enough with post-processing.

We gradually raised the temperature and observed that the center wavelength shifted to longer wavelengths with increasing temperature. The pitch of the LPFG is 540 μm, the spectrum of which is shown in Figure 3b (blue line). For temperatures above 700 °C, the center wavelength keeps shifting to longer wavelengths when the temperature increases, as shown in Figure 3a. Moreover, at higher temperatures, the residual stress releases faster. Thus, we raised the temperature to 750 °C and kept it constant for one hour, so that the residual stress could be quickly released. Then we lowered the temperature to 700 °C and kept it constant for two hours. We found that the center wavelength of the LPFG remained almost unchanged over 90 min. This means that the limit of residual stress releasing had been reached at 700 °C and there would not be any irreversible change below that temperature. Moreover, when the temperature dropped to room temperature, the center wavelength of the heated LPFG was not restored to the original wavelength of the unheated LPFG. In Figure 3b, from the comparison of the unheated and heated LPFG spectra, we found that the three resonance peaks shifted to longer wavelengths as a whole.

## 3. The High-Temperature Sensitivity of this LPFG

With Equation (1), the LPFG sensitivity to temperature can be derived [22], as shown in Equation (2):

(2) $$\frac{d\lambda }{dT}=\lambda \cdot \gamma \cdot \left(\alpha +\Gamma_{T}\right)$$

where α is the thermal expansion coefficient of the fiber (which is much smaller than Γ_T_), γ presents the part of the waveguide dispersion, the temperature dependence of the waveguide dispersion Γ_T_ can be expressed:

(3) $$\Gamma_{T}=\frac{\xi_{co}n_{fund,eff}-\xi_{cl}n_{cl,eff}}{n_{fund,eff}-n_{cl,eff}}$$

where the n_fund,eff_ and n_cl,eff_ is the effect of the refractive index of the fundamental mode and the cladding mode, respectively. The ξ_co_ and ξ_cl_ are the thermo-optic coefficients of the core and cladding, respectively. From Equation (3), if the difference between ξ_co_ and ξ_cl_ is greater, we can obtain a higher sensitivity to temperature. Since the thermo-optic coefficients of the GeO_2_ and SiO_2_ have a large difference, and the core of UHNA-7 is highly Ge-doped, we supposed that the LPFG on UHNA-7 could be sensitive to changes in temperature.

**Figure 4:**
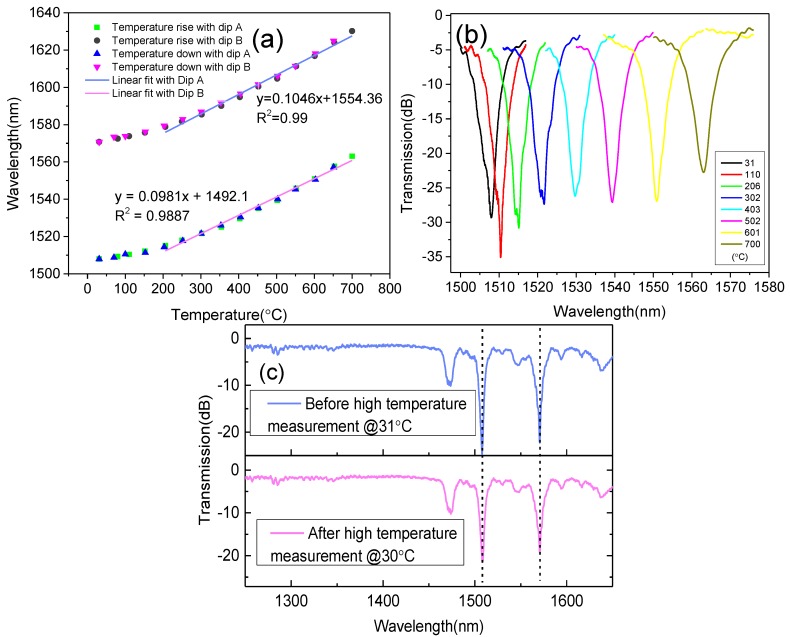
Variation of (**a**) the center wavelength of the LPFG and (**b**) its spectrum with temperature; and (**c**) the comparison with the spectra of the LPFG before and after high-temperature measurement.

We used an LPFG that had gone through heat-treatment, and presented the spectrum shown in Figure 3b for high temperature. We raised the temperature by 50 °C every 20 min and recorded the center wavelength (red points in Figure 4a). Then we lowered the temperature step-by-step and, again, recorded the center wavelength (blue points in Figure 4a). We saw that the heating and cooling curves matched perfectly, which means that the LPFG can be used for repeated measurements. The change in the spectrum of the LPFG with increasing temperature is shown in Figure 4b. Due to the high concentration of Ge in the core, the high-temperature sensitivity of this kind of LPFG is higher than some conventional LPFGs. The whole curve looks more quadratic than linear, since the thermo-optic coefficients of Ge are not linear [23]. If we perform a linear fit within a range from 200 °C to 700 °C, we obtain a high-temperature sensitivity for the LPFG of 0.0981 nm/°C (Dip B) and 0.1045 nm/°C (Dip A). The two dips have a similar sensitivity to high temperature. After the LPFG was cooled to room temperature again, we compared the spectra of the LPFG before and after being used for high-temperature measurements, as shown in Figure 4c. There is no obvious change in the spectrum from the comparison, which is different from the changing spectrum during the previous heat treatment in Figure 3b. This means that the LPFG is able to withstand high temperatures and be used as a high-temperature sensor.

**Figure 5:**
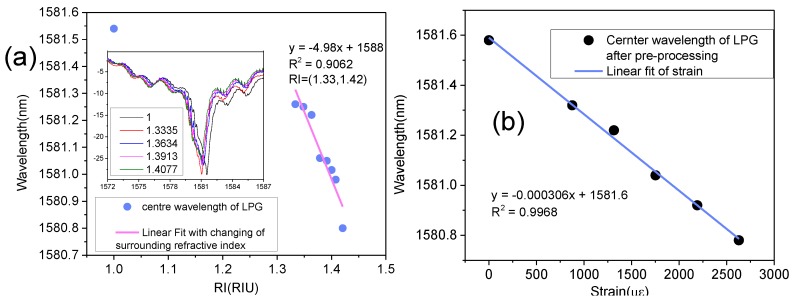
The LPFG’s sensitivity of (**a**) the environment refractive index; and (**b**) strain.

In addition, the LPFG’s response to the environment refractive index is shown in Figure 5a. The LPFG that is shown in Figure 5 has been used to measure high temperature. The RI sensitivity was –4.98 nm/RIU (RI is 1.333–1.420). When the surrounding RI changes from 1 to 1.42, the center wavelength of the LPFG only shifts within 0.8 nm, which is much smaller than the shift at high-temperature. Thus, we can say that this kind of LPFG is insensitive to RI, which is different from a conventional LPFG. The LPFG’s response to strain is shown in Figure 5b, the sensitivity is −0.306 pm/με, and the LPFG is not very sensitive to strain.

## 4. Discussion

**Figure 6:**
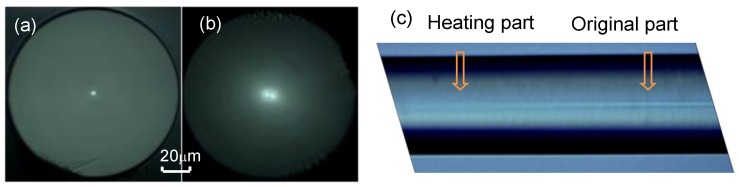
Cross-section of an (**a**) unheated and (**b**) heated UHNA-7 fiber, measured with a microscope. (**c**) Side image of LPFG which shows the heated and unheated part.

The compositions of the LPFG’s refractive index modulation come from two parts:

**The thermally-expanded core:** We used the CO_2_ laser to irradiate a part of the UHNA-7 fiber and make its core expand under high temperature. The power of the CO_2_ laser was 450 bits (15 W), the spot diameter of the CO_2_ laser was about 0.3 mm, and the scanning speed and range were 0.1 mm/s and 1 cm, respectively. After scanning it twice, we cut off the UHNA-7 fiber in the middle of the irradiated part and observed its cross-section with a microscope (see Figure 6b). Compared to the cross-section of the unheated UHNA-7 fiber shown in Figure 6a, we can see that there are two bright points that have resulted from the heated fiber, which present the high-order mode. The high-order mode has been excited, which means the diameter of the core has increased. In the side image of the LPFG (Figure 6c), we can find there is an obvious difference between the unheated and heated parts of the fiber. This also means the core has changed, which is caused by the diffusion of elements. The changed core will make the MFD change directly.

**Figure 7:**
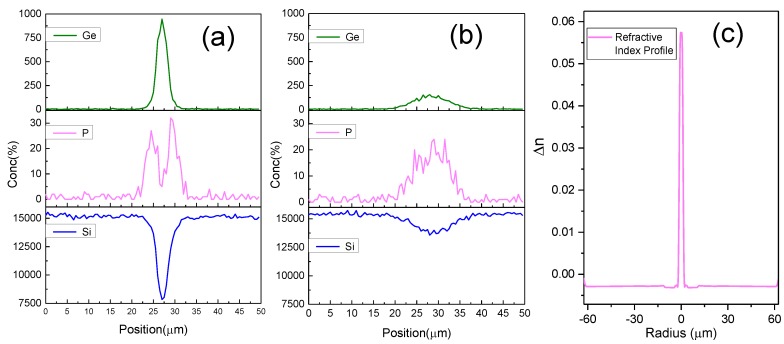
Element distribution on the cross-section of the (**a**) unheated and (**b**) heated UHNA-7 fiber. (**c**) The refractive index profile of the unheated UHNA-7 fiber.

**The Ge-diffusion of the core:** Furthermore, to understand exactly how the elements changed after heating, we measured the element distribution on the UNHA-7 fiber with an electron probe microanalyzer. The distributions of the three fiber elements (Si, P, and Ge) are depicted in Figure 7. Before heating with the CO_2_ laser, the Ge is concentrated in the core area and there is a P-doped layer around the core, as shown in Figure 7a. Hence, we can regard the UHNA-7 as a double-cladding fiber in which the core is highly doped with Ge, the first cladding is doped with P, and the second cladding is composed of pure silica. Since the UHNA-7 fiber has a very high NA, the Ge content is also very high, which causes the distribution of Si to present a hollow in the core area. After being heated with the CO_2_ laser, the Ge-doped area exhibited a significant change, as can be seen in Figure 7b. The diameter of this area increased from 4 μm to about 15 μm, and the peak of the Ge distribution curve decreased significantly, which means that the NA of the fiber decreased as well. By our qualitative calculation, the concentration of Ge in the processed fiber’s core dropped by over half in the fiber, which was before processing. This means that the effect on the RI change was on the order of 10^−2^ to 10^−3^. In a conventional LPFG, which is made by periodical tapering, the effect on the RI change caused by the changing of the fiber structure is on the order of 10^−4^ (unfortunately, due to equipment limitations, we are unable to obtain quantitative changes of Ge concentration at present).

The refractive index profile of the cross-section of the unheated UHNA-7 is shown in Figure 7c which is measured by an optical fiber analyzer (IFA-100, Interfiber Analysis, Sharon, MA, USA). From Figure 7c, we could find the refractive index (RI) of the core is much higher than the cladding, in which the difference of RI reaches 0.06 (the different RI of single-mode fiber (SMF-28) is about 0.003). In this respect, it also indicates that the doping content of Ge in the core is very high. Since the content of germanium in the core decreases, the core’s RI decreases obviously.

In conclusion, the modulation of LPFG on UHNA-7 comes from two parts: the expanded core (MFD) and the decreased core’s RI, which are both caused by the diffusion of elements.

## 5. Conclusions

In this study, we analyzed how the elements of a highly-doped fiber diffuse at high temperature. By controlling the diffusion of elements at high temperatures, we developed a technology of point-by-point heating and proposed a novel LPFG in a Ge/P-doped fiber. The modulation of this type of LPFG comes just from the periodic thermally expanded core, with no change in the cladding, which means it may be mechanically stronger than traditional LPFGs formed by tapering with a CO_2_ laser. The proposed LPFG can also withstand higher temperatures than LPFGs inscribed by a UV laser. Owing to the high Ge doping concentration, and to the large thermo-optic coefficients of this element, the LPFG has a high sensitivity at high temperature. However, due to limits in the range of BBS and OSA, we did not obtain the maximum temperature limit that the LPFG can withstand. Our further work will be focused on fabricating LPFGs with shorter center wavelengths, which have larger temperature measurement ranges.

## Figures and Tables

**Table 1:** The program for fabricating an LPFG on UHNA-7 fiber.

Special Function (Step 1)		Special Function (Step 2)	
Motor: Z-Left	Motor: Z-Right	Page 2	Page 7
Direction: Back	Direction: front	Power: 400 bits (special + 50 bits)	Main program: Jump
Start time: 0 ms	Start time: 0 ms	Start time: 0 ms	Next step: −1
Stop time: 3000 ms	Stop time: 3000 ms	Stop time: 9500 ms	Repeat number: 55
Speed: 0.2 μm/ms	Speed: 0.2 μm/ms		
